# An Inverse Problem for a Fractional Space–Time Diffusion Equation with Fractional Boundary Condition

**DOI:** 10.3390/e28010081

**Published:** 2026-01-10

**Authors:** Rafał Brociek, Agata Wajda, Christian Napoli, Giacomo Capizzi, Damian Słota

**Affiliations:** 1 Department of Artificial Intelligence Modelling, Faculty of Applied Mathematics, Silesian University of Technology, Kaszubska 23, 44-100 Gliwice, Poland; giacomo.capizzi@unict.it; 2 Department of Electrical, Electronics and Informatics Engineering, University of Catania, Viale Andrea Doria 6, 95125 Catania, Italy; 3 Institute of Energy and Fuel Processing Technology, Zamkowa 1, 41-800 Zabrze, Poland; awajda@itpe.pl; 4 Department of Computer, Control, and Management Engineering, Sapienza University of Rome, Via Ariosto 25, 00185 Roma, Italy; cnapoli@diag.uniroma1.it; 5 Department of Artificial Intelligence, Czestochowa University of Technology, Dabrowskiego 69, 42-201 Czestochowa, Poland; 6 Department of Mathematical Methods in Technology and Computer Science, Faculty of Applied Mathematics, Silesian University of Technology, Kaszubska 23, 44-100 Gliwice, Poland; damian.slota@polsl.pl

**Keywords:** inverse problem, time-space fractional diffusion equation, fractional boundary condition, identifying parameters

## Abstract

This article presents an algorithm for solving the direct and inverse problem for a model consisting of a fractional differential equation with non-integer order derivatives with respect to time and space. The Caputo derivative was taken as the fractional derivative with respect to time, and the Riemann–Liouville derivative in the case of space. On one of the boundaries of the considered domain, a fractional boundary condition of the third kind was adopted. In the case of the direct problem, a differential scheme was presented, and a metaheuristic optimization algorithm, namely the Group Teaching Optimization Algorithm (GTOA), was used to solve the inverse problem. The article presents numerical examples illustrating the operation of the proposed methods. In the case of inverse problem, a function occurring in the fractional boundary condition was identified. The presented approach can be an effective tool for modeling the anomalous diffusion phenomenon.

## 1. Introduction

Computer modeling plays a very important role in modern science. Many natural phenomena can be simulated using various types of mathematical models and computational techniques. One example is anomalous diffusion, which is described by a partial differential equation with a fractional derivative and initial–boundary conditions. Mathematical models of this type are used to describe many phenomena, such as heat flow in porous media [1], moisture transport modeled in terms of water head [2], and time-domain electromagnetic anomalous diffusion [3].

In modeling many processes, the use of fractional-order derivatives instead of integer-order derivatives often yields better results and proves to be effective. In [1], the authors compared mathematical models of heat conduction with fractional- and integer-order derivatives. The experiments presented in that paper show that models with fractional derivatives provide a better fit to the measurement data. Reference [4] presents a novel asymptotic stabilization control method for a fractional-order HIV-1 model. The mathematical model of the fractional-order HIV-1 infection includes state-space equations involving the Caputo fractional derivative. In turn, reference [5] presents a mathematical model of the dynamic behavior of tuberculosis. The authors report that, in their numerical simulations, the AB Caputo predictor–corrector approach exhibits improved performance compared to the classical fractional Caputo method for the considered tuberculosis model. Further applications of fractional calculus include the modeling of supercapacitors, batteries, a chain of vehicles operating in adaptive cruise control mode, and thermal processes inside buildings; see [6]. Reference [7] describes a cancer chemotherapy effect model involving fractional derivatives with an exponential kernel and the general Mittag–Leffler function. To solve the corresponding equations, the authors employ the Laplace homotopy perturbation method and the modified homotopy analysis transform method. The paper also presents numerical examples. In [8], the authors introduce operators of fractional derivatives along with their basic properties. Another interesting area in which fractional calculus has found applications is image encryption; an illustrative example can be found in [9].

For effective mathematical modeling, it is often necessary to solve both fractional and integer-order differential equations. There are many techniques available for solving this type of equation. In [10], Yu et al. present a numerical method for a multiterm time-fractional reaction–diffusion equation with classical Robin boundary conditions. The solution of the differential equation is a function of three variables, and the fractional derivative appearing in the equation is the Caputo derivative. The L1 finite difference method is used to construct the numerical scheme. In [11], an alternating direction implicit scheme is presented for solving a two-dimensional Riemann–Liouville distributed-order space-fractional diffusion equation. The authors prove that the proposed method is unconditionally stable and convergent. Numerical examples are also provided. In article [12], the authors present a solution of fractional differential equations using the homotopy analysis method (HAM). To determine the optimal parameters of the HAM, particle swarm optimization (PSO) is employed. The paper presents two examples in which nonlinear fractional-order differential equations with the Caputo derivative are solved.

In recent years, an increasing number of articles have addressed boundary value problems with fractional boundary conditions. For example, Baeumer et al. [13] considered, among others, fractional diffusion equations with fractional Neumann (reflecting) boundary conditions. The authors showed that the proposed model satisfies mass conservation. The well-posedness and steady-state solutions were also discussed in that paper. In turn, Kelly and co-authors [14] studied the two-sided space-fractional diffusion equation with Neumann boundary conditions. Explicit and implicit Euler methods were used to solve the problem. In [15], Xie and Fang presented two efficient numerical schemes for Riesz space-fractional diffusion equations with fractional Neumann boundary conditions. The authors applied the Crank–Nicolson method and the shifted Grünwald–Letnikov operator. The same authors, in [16], described the use of the Crank–Nicolson scheme for a space-fractional diffusion equation with Neumann or Robin fractional boundary conditions. In [17], the authors presented the use of a finite difference scheme to solve a time–space fractional diffusion equation with fractional boundary conditions (Neumann or Robin) imposed on one boundary of the considered domain. However, Jia and Wang [18] proposed a fast Krylov subspace iterative method for the efficient and accurate solution of systems of equations arising from finite difference methods, both steady-state and time-dependent, applied to one-dimensional space-fractional diffusion equations with fractional derivative boundary conditions. Examples of applications of models with fractional boundary conditions can be found in [19,20].

In many engineering applications, it is necessary to conduct computer simulations in order to design or effectively model certain phenomena. Problems of this type may involve the identification of model parameters, such as material, geometric, or other parametric data, and are commonly referred to as inverse problems. An example of such a problem is the estimation of aerothermal heating for a thermal protection system (TPS) [21,22]. The articles focus on the identification of heat flux in the TPS model, while the temperature is known at a control (measurement) point. An implicit finite difference scheme was used to solve the direct problem, whereas the inverse problem was solved using the Levenberg–Marquardt method. Referencee [23] addresses an inverse problem concerning the identification of thermal conductivity in transient heat transfer systems. The inverse problem was addressed using an improved artificial fish swarm optimization algorithm, demonstrating the effectiveness of swarm intelligence techniques for parameter identification in ill-posed heat transfer problems.

Inverse problems for fractional partial differential equations have also been extensively studied. Some of the earliest works in this area were published by Murio [24,25]. In these papers, the mollification method was used to reconstruct boundary conditions for fractional diffusion equations. Song and co-authors [26] considered the inverse problem of identifying the reaction coefficient for time-fractional diffusion equations in two-dimensional space. In turn, in [27], the source function was identified for a time-fractional diffusion–wave equation using the fractional Landweber method. In [28], the time-dependent heat coefficient in a one-dimensional time-fractional heat equation was reconstructed using a finite difference scheme combined with Tikhonov regularization. In [29], two inverse problems related to models with fractional derivatives were investigated. The model considered in that work was a two-dimensional generalized diffusion equation with a memory kernel. In the first case, a space-dependent source term was identified, while in the second problem a time-dependent source term was identified. In reference [30], an inverse problem for a differential equation with a Caputo fractional derivative was investigated. The study focused on reconstructing the function appearing on the right-hand side of the differential equation. The authors also presented theorems, together with proofs, concerning the existence and uniqueness of the solution. Liu et al. [31] consider the forward and inverse problems for a multiterm time-fractional Burgers-type equation. To solve both problems, they employ neural network–based methods, namely gradient-enhanced fractional physics-informed neural networks (gfPINNs), which constitute an improvement over standard fPINNs. The paper also includes a numerical example illustrating the accuracy of the proposed method. Reference [32] compared the use of various meta-heuristic algorithms to solve an inverse problem for a space-fractional heat conduction equation with a Riemann–Liouville fractional-order derivative. The compared algorithms include Ant Colony Optimization (ACO) for continuous functions, the Butterfly Optimization Algorithm (BOA), the Dynamic Butterfly Optimization Algorithm (DBOA), and Aquila Optimizer (AO). Among these methods, DBOA proved to be the most effective in terms of the value of the minimized objective function. Further examples of applications of inverse problems can be found in [33,34,35,36]. In all these articles, the authors considered classical integer-order boundary conditions. To the best of the authors’ knowledge, no existing work addresses inverse problems with fractional boundary conditions. The novelty of the present paper lies in the inclusion of a fractional Robin boundary condition in the formulation of the inverse problem.

This article presents the solution of both the direct and inverse problems for a fractional diffusion equation with a Robin-type fractional boundary condition. The novelty of this work lies in the identification of the function appearing in the fractional boundary condition based on additional measurement data. The inverse problem consists of determining the unknown function such that the resulting values of the state variable match the measurement data. To solve this problem, a metaheuristic algorithm called the Group Teaching Optimization Algorithm (GTOA) was employed. The paper also includes illustrative examples demonstrating the performance of the proposed algorithm.

### Main Contributions and Novelty

In recent years, fractional diffusion equations have been widely studied, including various inverse problems with fractional derivatives in time and space. Most existing research concentrates on identifying model parameters, determining the orders of fractional derivatives, or reconstructing source terms. These studies are usually conducted under classical boundary conditions, such as Dirichlet or Neumann conditions.

However, to the best of our knowledge, there are no existing studies that consider an inverse problem in which the unknown function appears in a fractional boundary condition involving a derivative of non-integer order on the boundary of the domain. Specifically, the presented model includes a fractional derivative in the boundary condition, which extends classical models and reflects more general anomalous interactions at the boundary (nonlocal effects). This type of boundary specification has not been previously used in numerical inverse problems for anomalous diffusion models. We propose a numerical algorithm based on a metaheuristic optimization algorithm (Group Teaching Optimization Algorithm, GTOA) for solving the inverse problem. In the inverse problem, we identify an unknown function appearing in the fractional boundary condition, which constitutes an additional layer of complexity absent in typical inverse problems in the literature. We provide computational evidence that the proposed approach effectively reconstructs boundary functions and exhibits robustness under measurement noise, indicating its practical applicability.

## 2. Preliminaries

This section is devoted to the basic concepts of fractional calculus, which are necessary for further reading of this article. The model presented in this article uses Caputo and Riemann–Liouville fractional derivatives.

**Definition 1.**  *Let f be a function that is piecewise continuous on the interval (a,+∞) and integrable on every finite subinterval of the [a,+∞), and let t>a and α>0. The Riemann–Liouville integral of non-integer order α has the following expression:*

(1) Dt−αaf(t)=1Γ(α)∫at(t−s)α−1f(s)ds.

Having defined the Riemann–Liouville integral operator of non-integer order, we introduce the concept of the Riemann–Liouville derivative of non-integer order.

**Definition 2.**  *Let the function f satisfy the assumptions given in the Definition 1, α>0 and n=α+μ, where ⌈α⌉=n. The Riemann–Liouville derivative of non-integer order α has the following expression:*

(2) DαaRLf(t)=DnDt−μaf(t)=1Γ(n−α)dndtn∫at(t−s)n−α−1f(s)ds,

*where $D^{n}$ denotes a classical derivative of integer order n.*

Now let proceed to defining and providing selected properties of the Caputo derivative.

**Definition 3.**  *The Caputo derivative of non-integer order α of the function f has the following expression:*

(3) DαaCf(t)=1Γ(n−α)∫atdnf(s)dsn(t−s)n−α−1ds,

*where α∈(n−1,n).*

The previous definitions are used to define partial derivatives of fractional order, used later in the whole paper.

The Caputo and Riemann–Liouville derivatives have the following relationship

(4) DαaRLf(t)=DαaCf(t)+∑k=0n−1(t−a)k−αΓ(k−α+1)dkf(a)dtk,

where t>a and α∈(n−1,n].

In addition to the Caputo and Riemann–Liouville derivatives, there are many other definitions of partial-order derivatives, for example the Grünwald–Letnikov, Riesz, or Weyl derivatives. Due to the fact that the Caputo and Riemann–Liouville derivatives are commonly used for describing the phenomena of heat transfer and diffusion, the following paragraphs focus on their description. More on non-integer order derivatives can be found in the books [37,38].

## 3. Fractional Model

In this article, we consider a model composed of a time–space fractional diffusion equation with fractional Robin boundary condition. In the remainder of this paper, we consider the Caputo fractional derivative with respect to time and the Riemann–Liouville fractional derivative with respect to space for the functions of two variables. Therefore, we adopt the notation $\frac{\partial^{\alpha }}{\partial t^{\alpha }}$ instead of DαaC, and $\frac{\partial^{\beta }}{\partial x^{\beta }}$ instead of DβaRL. Moreover, from Definitions 2 and 3, we assume that a=0; hence, *a* will no longer be explicitly indicated. This equation has the following form:

(5) $$\frac{\partial^{\alpha }u\left(x,t\right)}{\partial t^{\alpha }}=\lambda \left(x,t\right)\frac{\partial^{\beta }u\left(x,t\right)}{\partial x^{\beta }}+f\left(x,t\right),\;\;\;x\in \left(0,L\right),\;t\in \left(0,T\right],$$

with the initial condition

(6) u(x,0)=φ(x),x∈[0,L],

and boundary conditions

(7) u(0,t)=0,t∈(0,T],

(8) $$u\left(L,t\right)+{\left.\left(\lambda \left(x,t\right)\frac{\partial^{\beta -1}u\left(x,t\right)}{\partial x^{\beta -1}}\right)\right|}_{x=L}=\psi \left(t\right).$$

The boundary condition (8) defined on the right boundary is called the fractional Robin boundary condition. In Equation (5), the Caputo derivative defined by Formula (3) was assumed to be the fractional derivative with respect to time, and the Riemann–Liouville derivative (2) was assumed to be the fractional derivative with respect to space. We also assume that α∈(0,1),β∈(1,2), λ is a continuous, positive function called the diffusion coefficient, and the functions *f* (source term) and φ,ψ are also continuous functions.

Fractional boundary conditions arise in the modeling of anomalous diffusion processes, where nonlocal transport mechanisms occur not only in the bulk of the medium but also at its boundaries. In classical diffusion, the Robin boundary condition represents a balance between the concentration and the diffusive flux at the boundary and is commonly used to model semi-permeable, reactive, or partially absorbing boundaries. When anomalous diffusion is present, the classical flux operator is replaced by a fractional spatial derivative, leading, in a natural way, to a fractional Robin boundary condition. The fractional derivative of order β−1 appearing in the boundary condition can be interpreted as a nonlocal boundary flux, accounting for long-range interactions and memory effects at the boundary, which have been observed in transport processes in heterogeneous or porous media.

From a mathematical perspective, the fractional Robin boundary condition provides a natural closure condition for time–space fractional diffusion equations involving Riemann–Liouville derivatives in space. Such boundary conditions have been studied as generalizations of classical Robin conditions and ensure the well-posedness of the direct problem, similarly to the Dirichlet and Neumann conditions in the integer-order case. The inclusion of a fractional-order boundary operator reflects the nonlocal nature of the underlying spatial derivative and is consistent with the formulation of the governing equation.

In the next section, we present a numerical method to solve the problem (5)–(8).

## 4. Direct Problem

This section presents an implicit finite difference scheme for problem (5)–(8). The solution to this problem, i.e., finding the value of the *u* state function in the domain with known input values of the model, is called the solution of direct problem.

In order to describe the solution method of (5)–(8) we discretize the area [0,T]×[0,L], creating a mesh $S=\{\left(x_{i},t_{k}\right):i=0,1,\ldots ,N,\;k=0,1,\ldots ,K\}$, where

$$x_{i}=i\;\Delta x,\;i=0,1,\ldots ,N,\;\Delta x=\frac{L}{N},$$

$$t_{k}=k\;\Delta t,\;k=0,1,\ldots ,K,\;\Delta t=\frac{T}{K}.$$

The following notations have also been introduced:

$$\lambda_{i}^{k}=\lambda \left(x_{i},t_{k}\right),\;\;f_{i}^{k}=f\left(x_{i},t_{k}\right),\;\;\varphi_{i}=\varphi \left(x_{i}\right),\;\;\psi^{k}=\psi \left(t_{k}\right).$$

By $u_{i}^{k}=u\left(x_{i},t_{k}\right)$ we denote the values of the exact solution in points $\left(x_{i},t_{k}\right)$, and by $U_{i}^{k}$, the corresponding values obtained from the numerical solution.

In order to approximate the Riemann–Liouville derivative (2), we use the shifted Grünwald formula [39,40]:

(9) $${\left.\frac{\partial^{\beta }u\left(x,t\right)}{\partial x^{\beta }}\right|}_{\left(x_{i},t_{k+1}\right)}\approx \frac{1}{{\left(\Delta x\right)}^{\beta }}\sum_{j=0}^{i+1}g_{\beta ,j}U_{i-j+1}^{k+1},$$

where $g_{\beta ,j}=\frac{\Gamma (j-\beta )}{\Gamma (-\beta )\Gamma (j+1)}$. The Caputo derivative (3) with respect to time is approximated as follows [41]:

(10) $${\left.\frac{\partial^{\alpha }u\left(x,t\right)}{\partial t^{\alpha }}\right|}_{\left(x_{i},t_{k+1}\right)}\approx \frac{1}{\Gamma \left(2-\alpha \right){\left(\Delta t\right)}^{\alpha }}\sum_{j=1}^{k}\left(j^{1-\alpha }-{\left(j-1\right)}^{1-\alpha }\right)\left(U_{i}^{k-j+1}-U_{i}^{k-j}\right).$$

In our previous work [42], we tested the possibility of using the “short memory principle” [43,44] (treated in relation to the approximation of the fractional derivative, see [38]). The conclusion of that research was that the application of the “short memory principle” in the procedure significantly reduces the computation time, but the resulting solutions are affected by errors that cannot always be neglected. Since accuracy is of critical importance in solving the inverse problem, in the present study we chose not to apply the “short memory principle”.

The fractional derivative occurring in the boundary condition (8) is approximated as follows:

(11) $${\left.\frac{\partial^{\beta -1}u\left(x,t\right)}{\partial x^{\beta -1}}\right|}_{\left(x_{N},t_{k+1}\right)}\approx \frac{1}{{\left(\Delta x\right)}^{\beta -1}}\sum_{j=0}^{N+1}g_{\beta -1,j}U_{N-j+1}^{k+1}.$$

After taking into account the zero condition on the left boundary and using the Taylor expansion in the point $\left(x_{N+1},t_{k+1}\right)$, the approximation (11) can be written in the following form:

(12) $${\left.\frac{\partial^{\beta -1}u\left(x,t\right)}{\partial x^{\beta -1}}\right|}_{\left(x_{N},t_{k+1}\right)}\approx \frac{1}{{\left(\Delta x\right)}^{\beta -1}}\left(\sum_{j=1}^{N}g_{\beta -1,j}U_{N-j+1}^{k+1}+g_{\beta -1,0}\left(3U_{N}^{k+1}-3U_{N-1}^{k+1}+U_{N-2}^{k+1}\right)\right).$$

The convergence and stability of the finite difference approximations of the Caputo and Riemann–Liouville derivative operators are described in many works (e.g., [45,46]).

Substituting the approximations of fractional derivatives (9), (10) and the approximation of the boundary condition (12) into Equations (5)–(8), we obtain the following differential scheme:

- For i=1,2,…,N−1 and k=0
  (13) $$U_{i}^{1}-r\lambda_{i}^{1}\sum_{j=0}^{i}g_{\beta ,j}U_{i-j+1}^{1}=U_{i}^{0}+{\left(\Delta t\right)}^{\alpha }\Gamma \left(2-\alpha \right)f_{i}^{1},$$
- For i=1,2,…,N−1 and k=1,2,…,K−1
  (14) $$\begin{array}{cc} U_{i}^{k+1}\; & -r\lambda_{i}^{k+1}\sum_{j=0}^{i}g_{\beta ,j}U_{i-j+1}^{k+1}\\ \; & =\left(1-b_{1}\right)U_{i}^{k}+\sum_{j=1}^{k-1}\left(b_{j}-b_{j+1}\right)U_{i}^{k-j}+b_{k}U_{i}^{0}+{\left(\Delta t\right)}^{\alpha }\Gamma \left(2-\alpha \right)f_{i}^{k+1}. \end{array}$$
- For i=N and k=0,1,…,K−1
  (15) $$\begin{array}{cc} U_{N}^{k+1}\; & +\frac{\lambda_{N}^{k+1}}{{\left(\Delta x\right)}^{\beta -1}}\sum_{j=1}^{N}g_{\beta -1,j}U_{N-j+1}^{k+1}\\ \; & +\frac{\lambda_{N}^{k+1}g_{\beta -1,0}}{{\left(\Delta x\right)}^{\beta -1}}\left(3U_{N}^{k+1}-3U_{N-1}^{k+1}+U_{N-2}^{k+1}\right)=\psi^{k}. \end{array}$$
  where $b_{j}={\left(j+1\right)}^{1-\alpha }-j^{1-\alpha }$ and $r=\frac{{\left(\Delta t\right)}^{\alpha }\Gamma \left(2-\alpha \right)}{{\left(\Delta x\right)}^{\beta }}$. The difference scheme (13)–(15) can be written in matrix form as a system of equations in the following form:
  (16) $$A^{1}U^{1}=Q^{0}+F^{1},$$
  (17) $$A^{k+1}U^{k+1}=\left(1-b_{1}\right)Q^{k}+\sum_{j=1}^{k-1}\left(b_{j}-b_{j+1}\right)Q^{k-j}+b_{k}Q^{0}+F^{k+1},$$
  where the following vectors are denoted by $U^{k},Q^{k},F^{k}$:
  $$U^{k}={\left[U_{1}^{k},U_{2}^{k},\ldots ,U_{N}^{k}\right]}^{T},$$
  $$Q^{k}={\left[U_{1}^{k},U_{2}^{k},\ldots ,U_{N-1}^{k},0\right]}^{T},$$
  $$F^{k}=\left[{\left(\Delta t\right)}^{\alpha }\Gamma \left(2-\alpha \right)f_{1}^{k},{\left(\Delta t\right)}^{\alpha }\Gamma \left(2-\alpha \right)f_{2}^{k},\ldots ,{\left(\Delta t\right)}^{\alpha }\Gamma \left(2-\alpha \right)f_{N-1}^{k},{\left(\Delta x\right)}^{\beta -1}\psi^{k}\right].$$
  The symbol $A^{k}$ denotes the matrix of dimension N×N, whose coefficients for the time index k=0.1,…,K−1 are given by the following formula:
  (18) $$a_{ij}^{k}=\left\{\begin{array}{cc} -r\lambda_{i}^{k+1}g_{\beta ,i-j+1}, & 1\leq j\leq i-1,\;1\leq i\leq N-1,\\ 1-r\lambda_{i}^{k+1}g_{\beta ,1}, & 1\leq j=i\leq N-1,\\ -r\lambda_{i}^{k+1}g_{\beta ,0}, & j=i+1,\;1\leq i\leq N-1,\\ \lambda_{N}^{k+1}g_{\beta -1,N-j+1}, & 1\leq j\leq N-3,\;i=N,\\ \lambda_{N}^{k+1}g_{\beta -1,3}+\lambda_{N}^{k+1}g_{\beta -1,0}, & j=N-2,\;i=N,\\ \lambda_{N}^{k+1}g_{\beta -1,2}-3\lambda_{N}^{k+1}g_{\beta -1,0}, & j=N-1,\;i=N,\\ {\left(\Delta x\right)}^{\beta -1}+\lambda_{N}^{k+1}g_{\beta -1,1}+3\lambda_{N}^{k+1}g_{\beta -1,0}, & j=i=N,\\ 0, & i+2\leq j\leq N,\;1\leq i\leq N-2. \end{array}\right.$$

**Figure 1:**
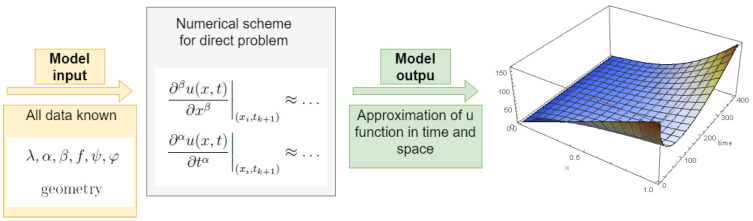
Scheme of solution for the direct problem.

For fixed grid sizes and fixed time t=kΔt, by solving the system of Equations (16) or (17) we will obtain approximate values in grid points of the solution state function *u* in Equation (5). Schematically, the process of solving the direct problem is shown in Figure 1. More on the convergence of the presented differential scheme can be found in [17].

## 5. Inverse Problem

If all data of model (5)–(8) are known, solving the direct problem using the method described in Section 4 will provide us with the temperature distribution in time and space. In many engineering problems, some of the data is unknown. This may result from difficulties related to measurements or the initial design stage, where material, geometric data, or boundary conditions must be determined so that the designed process runs in accordance with the assumptions. The situation where part of the model data is unknown and the data must be selected appropriately is called the inverse problem.

**Figure 2:**
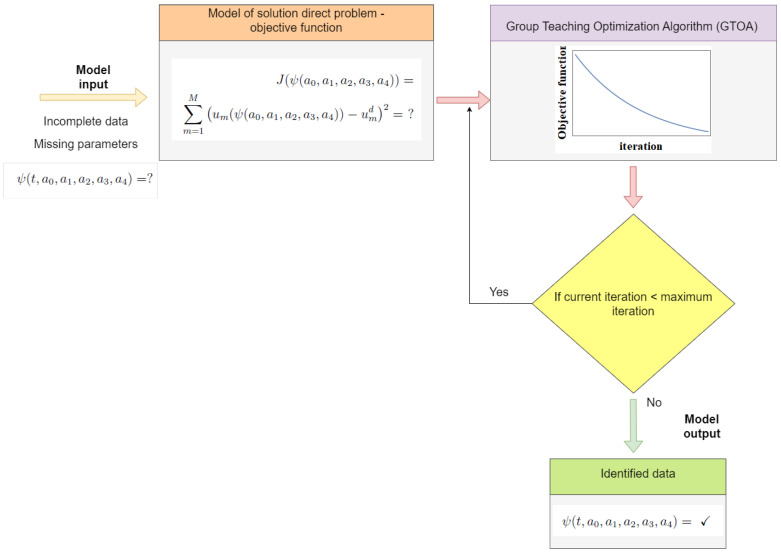
Scheme of the solution for the inverse problem.

In the inverse problem considered in this article, function ψ, describing the boundary condition of the third type occurring on the right boundary of the considered domain, is unknown. In the presented model, we are dealing with the fractional boundary condition. The unknown function ψ is dependent on five numerical coefficients $a_{0},a_{1},a_{2},a_{3},a_{4}$, so we can write $\psi (t,a_{0},a_{1},a_{2},a_{3},a_{4})$. Details about the form of the unknown function ψ are described in the example in Section 6.2. In order to properly identify the sought model parameters, additional information regarding the process is needed. In thermal issues, this additional information is often temperature measurements (values of the state function *u*) in measurement points (or control points), hereinafter referred to as measurement data. Then, the unknown parameters should be selected so that the difference between the measurement data and the values obtained from the model is as small as possible. For this purpose, a fitness function is built depending on unknown parameters, which in our case has the following form:

(19) $$J\left(\psi \left(a_{0},a_{1},a_{2},a_{3},a_{4}\right)\right)=\sum_{m=1}^{M}{\left(u_{m}\left(\psi \left(a_{0},a_{1},a_{2},a_{3},a_{4}\right)\right)-u_{m}^{d}\right)}^{2},$$

where the searched numerical coefficients $a_{0},a_{1},a_{2},a_{3},a_{4}$ define the function ψ, *M* is the number of measurement data, $u_{m}$ means the values of the function *u* in control points obtained from the model, and $u_{m}^{d}$ denotes the measurement data. The considered problem comes down to effectively searching for the minimum of the fitness function (19). It should be noted here that we do not have an analytical formula for this function; we can only calculate its value in a given point in the search space. Therefore, classic optimization methods cannot be used. The second problem in solving this inverse problem is the time needed to calculate the value of the *J* function. A single calculation of the value of this function requires solving a direct problem, and therefore many systems of equations. Hence, it is extremely important that the optimization method of searching the minimum works with the fewest number of calls to the objective function. In this article, a metaheuristic called the Group Teaching Optimization Algorithm (GTOA) [47] is used to search for the minimum of the function (19). The diagram of the algorithm for identifying unknown parameters in the inverse problem is presented in Figure 2.

### Group Teaching Optimization Algorithm

The described algorithm was inspired by the mechanisms governing the learning process in a group of students. The idea of the proposed GTOA is to improve the knowledge of the entire class (group of students) by simulating the mechanism of group teaching. Many multi-agent algorithms of this type can be found in the scientific literature. Depending on the problem being solved, various algorithms of this type may work better or worse, and the range of their applications is wide [23,32,48,49,50].

GTOA is inspired by the process of students learning in groups, either among themselves or with the help of a teacher. In the assumptions of the described algorithm, we can distinguish four main mechanisms:

- The ability to absorb knowledge determines the division of students into two groups—average and outstanding,
- The teacher should pay more attention to weaker students than to better ones, so they should use different techniques for different groups of students,
- Students can learn in their free time, either by developing their skills or by interacting with other students,
- A good teacher allocation mechanism is very helpful in improving students’ knowledge.

In GTOA, we can distinguish four main stages: teacher allocation phase, ability grouping phase, teacher phase and student phase. Each stage is described below, providing the appropriate mathematical formulas necessary to implement the algorithm.

**Figure 3:**
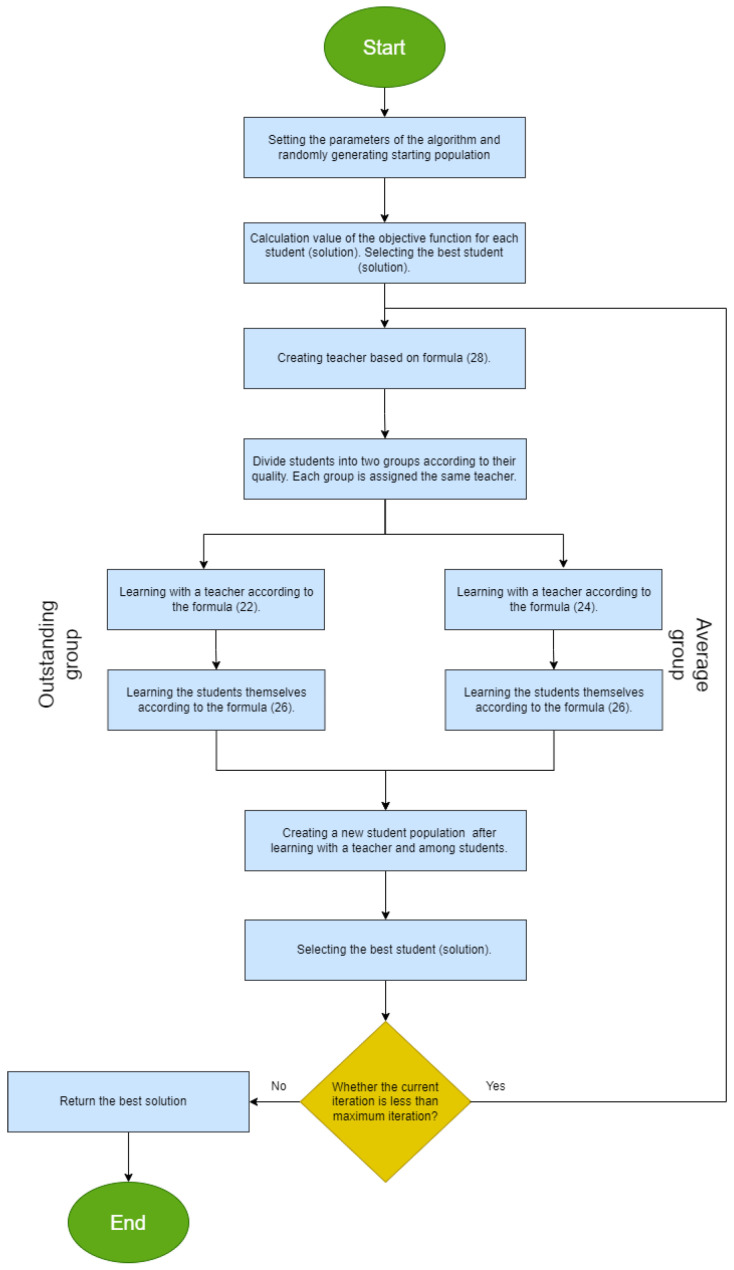
Block diagram for GTOA.

- Ability Grouping Phase: In accordance with the assumptions of the algorithm and the idea of good group learning, it is worth dividing large groups of students into smaller ones depending on their abilities. Then, within the groups, there will be little variation in students’ knowledge, which has a positive impact on the learning process. In the case of the described algorithm, we divide the entire group of students into two equal groups—one average, the other outstanding. In each iteration, a process of dividing students is necessary. Each group is assigned a different teacher, which is reflected in the algorithm in the fact that each group has different learning parameters/mechanisms.
- Teacher Phase: In the case of an outstanding group, the main goal is to increase the average knowledge level of this group. Through translating this principle into a mathematical description, we obtain the following formula:
  (20) $$x_{teacher,i}^{k+1}=x_{i}^{k}+a\left(T^{k}-F\left(bM^{k}+cx_{i}^{k}\right)\right),$$
  where $x_{teacher,i}^{k+1}$ means *i*th student after the teaching phase, $x_{i}^{k}$ is the *i*th student before learning with teacher, $T^{k}$ is the teacher’s knowledge, and $M^{k}$ is the mean knowledge of the group, defined by the following formula:
  (21) $$M^{k}=\frac{1}{N}\sum_{i=1}^{N}x_{i}^{k}.$$
  Parameters a,b,c are random numbers in the range [0,1], and *F* is called the teaching factor. Typically, its value is 1 or 2 [47].
  In the case of a group of students with average knowledge, the teacher uses different teaching techniques. Mathematically, this is reflected in the following formula:
  (22) $$x_{teacher,i}^{k+1}=x_{i}^{k}+2d\left(T^{k}-x_{i}^{k}\right),$$
  where *d* is a random number in the range [0,1]. In the case of both groups, the solution that goes to the next stage is either $x_{teacher,i}^{k+1}$ or $x_{i}^{k}$, depending on which solution obtained the lower objective function value
  (23) $$x_{teacher,i}^{k+1}=\left\{\begin{array}{cc} x_{teacher,i}^{k+1}, & \mathrm{for}\;J\left(x_{teacher,i}^{k+1}\right)<J\left(x_{i}^{k}\right),\\ x_{i}^{k}, & \mathrm{for}\;J\left(x_{teacher,i}^{k+1}\right)\geq J\left(x_{i}^{k}\right). \end{array}\right.$$
- Student Phase. After the stage of working with the teacher, there comes the stage of working with the student. In their free time, students can also improve their skills and expand their knowledge. They can do this in two ways: through self-teaching or through contact with other students. In the algorithm, this process is described by the following formula:
  (24) $$x_{student,i}^{k+1}=\left\{\begin{array}{c} \begin{array}{cc} x_{teacher,i}^{k+1}\; & +e\left(x_{teacher,i}^{k+1}-x_{teacher,j}^{k+1}\right)+g\left(x_{teacher,i}^{k+1}-x_{i}^{k}\right)\\ \; & \;\mathrm{for}\;J\left(x_{teacher,i}^{k+1}\right)<J\left(x_{teacher,j}^{k+1}\right), \end{array}\\ \begin{array}{cc} x_{teacher,i}^{k+1}\; & -e\left(x_{teacher,i}^{k+1}-x_{teacher,j}^{k+1}\right)+g\left(x_{teacher,i}^{k+1}-x_{i}^{k}\right)\\ \; & \;\mathrm{for}\;J\left(x_{teacher,i}^{k+1}\right)\geq J\left(x_{teacher,j}^{k+1}\right). \end{array} \end{array}\right.$$
  Formula (24) has the following notations: $x_{student,i}^{k+1}$—*i*th student after student phase; $x_{teacher,i}^{k+1}$—*i*th student after learning with teacher; $x_{i}^{k}$—*i*th student before learning with teacher; e,g are random numbers from interval [0,1]; *J* is the objective function. In this formula, the student with index *j* is another random student; hence, j∈{1,2,…,i−1,i+1,…,N}. Those students who improved after the self-learning stage move to the next iteration
  (25) $$x_{i}^{k+1}=\left\{\begin{array}{cc} x_{teacher,i}^{k+1}, & \mathrm{for}\;J\left(x_{teacher,i}^{k+1}\right)<J\left(x_{student,i}^{k+1}\right),\\ x_{student,i}^{k+1}, & \mathrm{for}\;J\left(x_{teacher,i}^{k+1}\right)\geq J\left(x_{student,i}^{k+1}\right). \end{array}\right.$$
- Teacher Allocation Phase: In this step, teacher $T^{k}$ is determined. In the proposed algorithm, this is achieved according to the following formula:
  (26) $$T^{k}=\left\{\begin{array}{cc} x_{best}^{k}, & \mathrm{for}\;J\left(x_{best}^{k}\right)<J\left(\frac{x_{best}^{k}+x_{second}^{k}+x_{third}^{k}}{3}\right),\\ \frac{x_{best}^{k}+x_{second}^{k}+x_{third}^{k}}{3}, & \mathrm{for}\;J\left(x_{best}^{k}\right)\geq J\left(\frac{x_{best}^{k}+x_{second}^{k}+x_{third}^{k}}{3}\right), \end{array}\right.$$
  where the indexes best, second, and third denote the three best solutions in the entire population. Once you have a full description of the algorithm, along with its formulas, you can start implementing it. Figure 3 shows the block diagram for GTOA.

## 6. Numerical Example

In the following examples, when calculating errors in the approximate solution for direct problems, we used the following formula for mean and maximum error:

(27) $$E_{mean}=\frac{1}{N\times K}\sum_{i=0}^{N}\sum_{k=0}^{K}\left|u_{e}\left(x_{i},t_{k}\right)-u_{a}\left(x_{i},t_{k}\right)\right|$$

(28) $$E_{max}=\max_{i,k}\left|u_{e}\left(x_{i},t_{k}\right)-u_{a}\left(x_{i},t_{k}\right)\right|$$

where $u_{e}$ is an exact solution and $u_{a}$ is an approximate solution. First, we present a numerical example for solving the direct problem, then an example for identifying fractional boundary conditions in an anomalous diffusion model.

### 6.1. Example of Solution Direct Problem

In this example, a direct problem with the following data is considered:

α=0.6,β=1.5,x∈[0,1],t∈[0,10],λ(x,t)=2xt,

and the initial condition and the condition on the right boundary are as follows:

$$u\left(x,0\right)=x^{3},$$

$$u\left(1,t\right)+{\left(\lambda \left(x,t\right)\left.\frac{\partial^{\beta -1}u\left(x,t\right)}{\partial x^{\beta -1}}\right)\right|}_{x=1}=1+t+t^{2}+\frac{4t(8+5t+5t^{2})}{5\sqrt{\pi }}.$$

The additional term on the right side of the Equation (5) is the following function:

$$f\left(x,t\right)=-\frac{2t\sqrt{x}\left(t+t^{2}+8x^{2}\right)}{\sqrt{\pi }}+\frac{5t^{0.4}x\left(7+10t\right)}{14\Gamma (0.4)}.$$

For the presented data, the exact solution is known $u\left(x,t\right)=x\left(t+x^{2}+t^{2}\right)$. All numerical computations were carried out in double precision using a fully self-implemented code written in C#. The simulations were performed on a desktop computer equipped with an Intel(R) Core(TM) i7-8550U CPU (1.80 GHz) and 20 GB RAM, running a 64 bit Windows 10 operating system. At each time step, a linear system of algebraic equations arising from the spatial discretization is assembled and solved. In the present implementation, the resulting linear systems are solved using a direct Gaussian elimination method.

**Figure 4:**
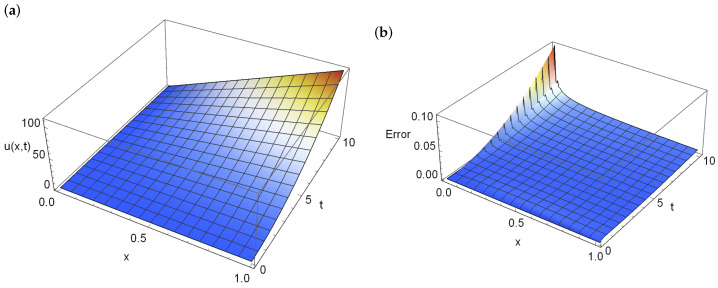
Graph of the approximate solution (**a**) and errors of this approximation (**b**) in the entire domain for a mesh of dimension 100×100.

**Figure 5:**
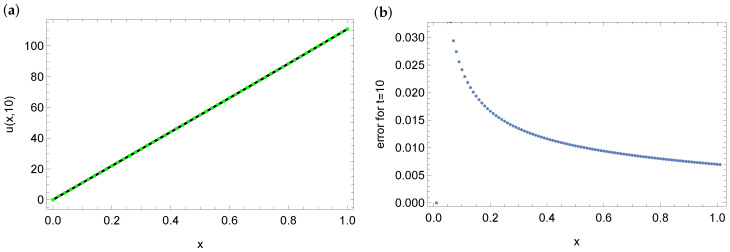
Graph of the approximate solution (green dots) and the exact solution (black line) (**a**) and errors of this approximation (**b**) for t=10 and mesh size 100×100.

Table 1 shows the mean and maximum errors of approximation and computation time depending on the size of the used mesh. For the 10×10 mesh, the mean error is $10^{-1}$, while resizing the mesh to 500×500 reduced the mean error to $10^{-5}$. We can also see that increasing the mesh size relative to space reduces the errors, while increasing the mesh size over time does not significantly reduce the error. Figure 4 presents the approximate solution (left part of the figure) and the errors of this approximation (right part of the figure) for the entire domain in the case of calculations for a 100×100 grid. Figure 5 shows the exact and approximate solution (*a*) and the error of this solution for the final moment t=10 (*b*). As can be seen in both figures, the largest errors are on the left side of *x*.

To assess the convergence properties of the proposed numerical scheme for the direct problem, the observed orders of accuracy were computed based on the mean error $E_{\mathrm{mean}}$ (see Table 1). For two successive meshes with characteristic mesh sizes $h_{1}$ and $h_{2}$, the convergence order *p* was estimated using the standard formula

(29) $$p=\frac{\log\;\left(E\left(h_{1}\right)/E\left(h_{2}\right)\right)}{\log\;\left(h_{1}/h_{2}\right)},$$

where E(h) denotes the corresponding numerical error.

The numerical results (see Table 2 and Table 3) indicate approximately first-order convergence with respect to the spatial discretization, while refining the temporal mesh alone does not significantly improve the accuracy. This suggests that the overall error is dominated by the spatial discretization.

**Figure 6:**
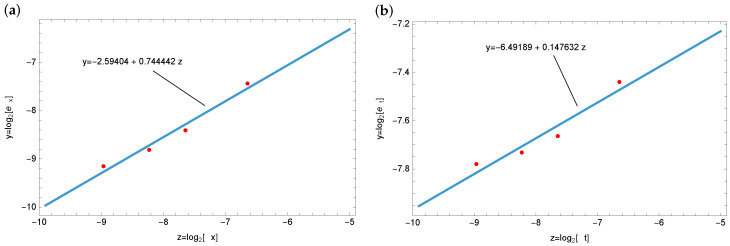
Empirical evaluation of the convergence order with respect to the spatial variable (**a**), and with respect to the time variable (**b**).

Figure 6 presents an empirical assessment of the convergence behavior. For the spatial variable, an approximate convergence rate of 0.74 is observed, whereas for the temporal variable, the estimated rate is close to 0.14. The theoretical orders are expected to be valid only for sufficiently small step sizes Δx and Δt. Hence, the discrepancies between the theoretical predictions and the observed estimates can most likely be attributed to relatively large discretization steps and the resulting approximation errors.

### 6.2. Inverse Problem–Benchmark Example

This section presents a computational example of the inverse problem using the algorithm described in Section 5. In the considered inverse problem, the fractional boundary condition (8) on the right boundary is unknown and must be identified. In general, these types of inverse problems are considered difficult to solve and require special attention. The following numerical data is assumed in the (5)–(8) model:

x∈[0,1],t∈[0,400],α=0.6,β=1.5,λ(x,t)=2xt,φ(x)=150x,

$$f\left(x,t\right)=\frac{5t^{2/5}x^{2}}{2\Gamma \left(\frac{2}{5}\right)}-\frac{10\sqrt{\pi }x^{3/2}}{\sqrt[{10}]{t}\Gamma \left(\frac{9}{10}\right)}-\frac{2t\sqrt{x}\left(4tx-15\pi \sqrt{tx}+150\right)}{\sqrt{\pi }}.$$

The fractional boundary condition (8) is given by the function ψ. This example (benchmark) is intended to test the proposed algorithm; hence, the sought function ψ is known and has the following form:

$$\psi \left(t\right)={\left(\sqrt{t}-10\right)}^{2}+\frac{2t\left(8t-45\pi \sqrt{t}+900\right)}{3\sqrt{\pi }}+50.$$

Writing the ψ function in numerical form, the following is obtained:

$$\psi \left(t\right)=3.00901t^{2}-53.1736t^{3/2}+339.514t-20t^{1/2}+150.$$

Hence, the sought function ψ has the following form:

(30) $$\psi \left(t\right)=a_{0}\;t^{2}+a_{1}\;t^{3/2}+a_{2}\;t+a_{3}\;t^{1/2}+a_{4},$$

where parameters $a_{0},a_{1},a_{2},a_{3},a_{4}$ are unknown. The following search space is adopted in the task: $a_{0}\in \left[0,6\right]$, $a_{1}\in \left[-80,-20\right]$, $a_{2}\in \left[200,500\right]$, $a_{3}\in \left[-40,0\right]$ and $a_{4}\in \left[0,300\right]$. The measurement data for the inverse problem are the values of the *u* state function collected from three separate control points $x_{p_{1}}=1$ (right edge), $x_{p_{2}}=0.8$ and $x_{p_{1}}=0.5$ (center). This data is obtained as a result of solving the direct problem for a grid size of 400×400. While the algorithm of the inverse problem is running, a grid size of 100×200 is assumed. The measurement data were also distorted by pseudo-random errors of 1%, 5%, and 10%. This approach aims to test the proposed algorithm and examine its stability depending on the accuracy of the measurement data as well as the location of the measurement point.

GTOA, described in Section 5, is used to search for the minimum of the functional (19) describing the error of the approximate solution. Due to the fact that the described algorithm belongs to the group of probabilistic methods, it was decided to repeat the calculations for each case five times. Moreover, the following parameters were adopted in GTOA: population_size =40; number_of_iteration =70. As a result of the calculations, it is observed that number_of_iteration could be reduced in many cases without any significant harm to the final result. However, the value of 70 used in the examples guaranteed the stability of the obtained results, as evidenced by the low value of standard deviation in Table 4.

Table 4 presents the identified values of unknown parameters $a_{0},\ldots ,a_{4}$ for the control point $x_{p}=1$. In most cases, they are close to the reference values. The relative errors of identification $a_{0},a_{1},a_{2}$ are very low. The largest errors were obtained for the coefficient $a_{3}$, but it should be mentioned here that the last two coefficients $a_{3},a_{4}$ have the smallest impact on the identified function ψ (30). As the disturbances of the measurement data increase, in general, the reconstruction results are slightly worse, although the exception is the case of disturbances with the 5% error, where the errors turned out to be the highest. However, in this case, the errors are also at a satisfactory level. It is also natural to increase the value of the objective function *J* if the disturbance of the measurement data increases.

Another indicator of the quality of the obtained solution is the errors of estimation ψ function occurring in the boundary condition. These errors are calculated from the following formulas:

(31) $$\begin{array}{cc} E_{abs} & =\frac{1}{t^{*}}\;\int_{0}^{t^{*}}\left|\psi_{ex}\left(t\right)-\psi_{est}\left(t\right)\right|\;dt, \end{array}$$

(32) $$\begin{array}{cc} E_{rel} & =E_{abs}\;{\left(\frac{1}{t^{*}}\;\int_{0}^{t^{*}}\left|\psi_{ex}\left(t\right)\right|\;dt\right)}^{-1}\;\times 100\%, \end{array}$$

where $\psi_{ex}$ is the exact value of ψ function and $\psi_{est}$ denotes the estimated ψ, while $t^{*}$ is the final time, which in the example is 400. The relative errors in reproducing the ψ are minimal (see the results in Table 5). For the largest disturbances of 10% of the measurement data, the relative error is 0.108%.

**Figure 7:**
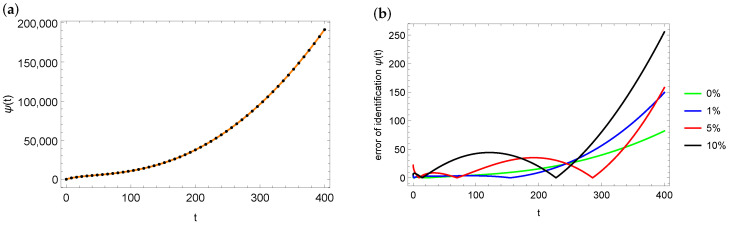
Exact (orange solid line) and estimated (black dots) function ψ for the case of 10% noise in measurement data and $x_{p}=1$ (**a**), errors distribution of ψ estimation depending on noise in the measurement data for $x_{p}=1$ (**b**) for mesh 100×200.

Figure 7a shows the plot of the exact ψ function (marked with the orange solid line) and the identified value of the ψ function for data perturbed by the 10% error (black dots). As can be seen in Figure 7a, the fit is very good. The situation is similar in the case of other input data disturbances. Figure 7b presents the distribution of errors in reproducing the ψ function depending on the disturbances in the measurement data. The nature of this distribution is similar in each case, with the largest errors at the end of the considered interval.

**Figure 8:**
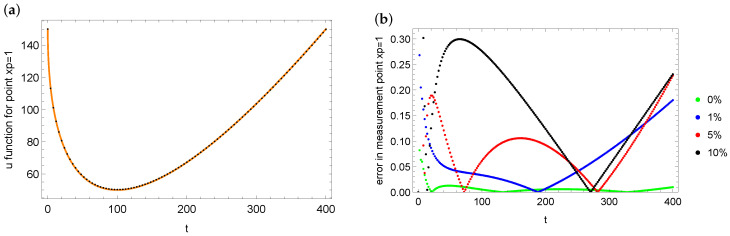
Fit of the identified values of the *u* function (black dots) to the measurement data (orange dots) in the measurement point $x_{p}=1$ (case 10% noise): (**a**) errors in the reconstruction *u* function in measurement point $x_{p}=1$ for different noise levels in the measurement data (**b**) for mesh 100×200.

Another indicator of the quality of the obtained solution is the impact of the ψ reconstruction errors on the values of the *u* function at the measurement point—in other words, how, after substituting the identified boundary condition into the model, the values of the *u* state function at the measurement point are adjusted to the measurement data. Figure 8a shows the exact (orange dots) and reconstructed (black dots) values of the *u* state function in the control point $x_{p}=1$ in the case of data disturbed by the 10% error. Figure 8b presents errors distribution in measurement point $x_{p}=1$ for identified boundary condition depending on different noise level of measurement data. The results are consistent with expectations, i.e., the smallest errors are for accurate measurement data (undisturbed by errors), and the greater the noise in the data, the greater the error when reproducing these data. However, it should be emphasized that, in each case, these errors are small and the results obtained are satisfactory.

The next step in the research is to test the algorithm by changing the position of the measurement point. For this purpose, we changed the control point to $x_{p}=0.8$ and $x_{p}=0.5$ (in the middle of the considered area), while all other aspects of the experiment remained unchanged. The results restored the parameters $a_{0},\ldots ,a_{4}$ to a similar level as in the case of the measurement point $x_{p}=1$. Also, in the case of other control points, the largest errors were for the coefficient $a_{3}$ and the smallest for $a_{0}$, as shown in Table 6 and Table 7. In Table 7, you can see large errors in the identification of $a_{3}$ for the case of 5% noise. Another significant difference is the value of the objective function. The closer the measurement point is to the center of the considered domain, the smaller the value of the objective function. For example, for measurement data distorted by an error of 10%, the values of the objective function for measurement points 1, 0.8, and 0.5, respectively, are approximately 1681, 703, and 201. It can be said that the presented algorithm is resistant to changes in the location of the measurement point.

Table 8 shows the ψ function identification errors. In all but one case, the relative errors were less than 0.2%. Only in the case of point $x_{p}=0.5$ and 5% noise in the measurement data did the relative error exceed 2%, which is a lower value than the noise in the measurement data.

**Figure 9:**
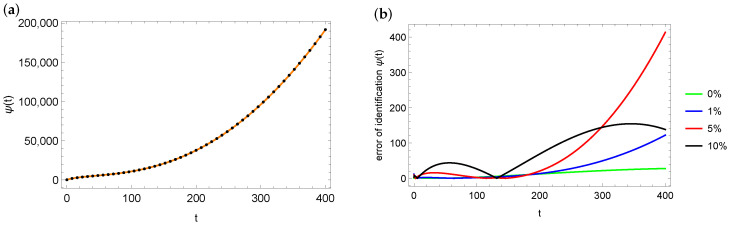
Exact (orange solid line) and estimated (black dots) function ψ for the case of 10% noise in measurement data and $x_{p}=0.8$: (**a**) error distribution of ψ estimation depending on noise in measurement data for $x_{p}=0.8$ (**b**) for mesh 100×200.

**Figure 10:**
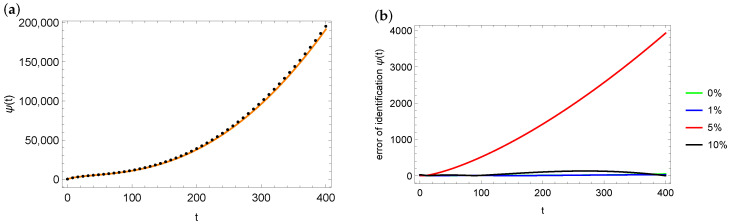
Exact (orange solid line) and estimated (black dots) function ψ for the case of 5% noise in measurement data and $x_{p}=0.5$: (**a**) error distribution of ψ estimation depending on noise in measurement data for $x_{p}=0.5$ (**b**) for mesh 100×200.

Similarly to the description of the results obtained for control point $x_{p}=1$, Figure 9 and Figure 10 present graphs of the identified functions ψ (part (a)) and the errors in this identification (part (b)) for measurement points $x_{p}=0.8$ and $x_{p}=0.5$. As in the case of $x_{p}=1$, the largest errors are found at the end of the considered time interval. A noticeable difference is the significantly larger maximum error in the case of noisy data with the 5% error. However, if we look at Table 5 and Table 8 and compare the relative errors for this case (measurement points $x_{p}=1$ and $x_{p}=0.8$), these differences are not significant—0.055% for $x_{p}=1$ and 0.153% for $x_{p}=0.8$. In the case of measurement point $x_{p}=0.5$ and 5% noise, the reconstruction error of ψ is clearly larger than in any other case. These figures confirm the data contained in Table 8.

**Figure 11:**
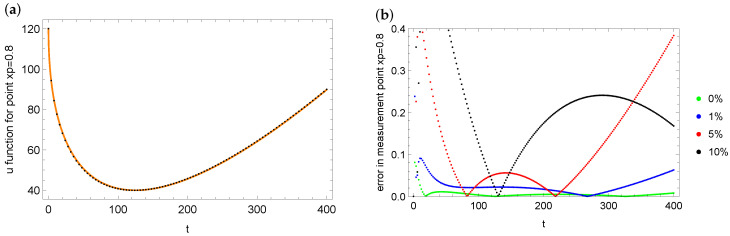
Matching the identified values of the *u* function (black dots) to the measurement data (orange dots) at measurement point $x_{p}=0.8$ (case of 10% noise): (**a**) errors in reconstruction *u* function in measurement point $x_{p}=0.8$ for measurement data with different noise levels (**b**) for mesh 100×200.

**Figure 12:**
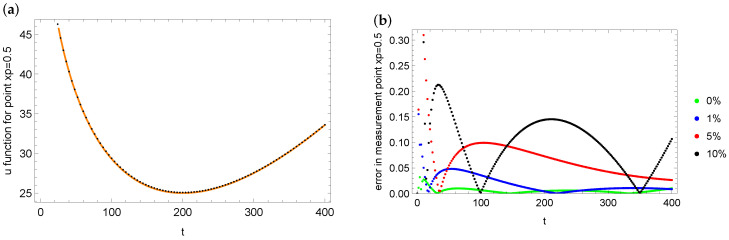
Matching the identified values of the *u* function (black dots) to the measurement data (orange dots) at measurement point $x_{p}=0.5$ (case of 5% noise): (**a**) errors in reconstruction *u* function at measurement point $x_{p}=0.5$ for measurement data with different noise levels (**b**) for mesh 100×200.

In the end of this example, we also present the errors in reconstruction of the *u* state function and its fit with the measurement data. Figure 11 shows the match between the reconstruction of the *u* function at measurement point $x_{p}=0.8$ in the case of noise with error 10% (a) and the reconstruction errors of *u* for various measurement data with different levels (b). It can be seen that the largest errors of fit with the measurement data in the case of the point $x_{p}=0.8$ are obtained for the noise level 5%. The smallest errors are obtained for exact measurement data. Analogous plots for the case $x_{p}=0.5$ and noise 5% are presented in Figure 12. In all cases, the fit of the identification *u* state function to the measurement data is very good.

## 7. Conclusions

In this article, we presented a numerical approach to solve direct and inverse problems for a fractional time-space differential equation with a fractional Robin boundary condition. The finite difference scheme was used to solve the direct problem, and the GTOA metaheuristic method was used to solve the inverse problem. The algorithm for the inverse problem presented in this paper was described step by step and tested on an example, and the fractional boundary condition was identified. The obtained results showed that the algorithm reconstructs the sought values well, and the errors in the reconstructed measurement data were small. The algorithm was also tested for its sensitivity to the location of the measurement point.

As the measurement point moves away from the boundary where the condition is identified, the reconstruction errors increase slightly. This indicates a limitation of the method: the accuracy of reconstruction depends on the proximity of the measurement point to the boundary. Nevertheless, in all cases, the errors in identifying the ψ function remained smaller than the input data errors.

The presented approach can be an effective tool for modeling anomalous diffusion phenomena. Furthermore, the computational cost of the metaheuristic algorithm may become significant for larger or more complex problems; this could be mitigated by employing parallel computing or other algorithmic optimizations. Future work could explore multiple measurement points, more complex boundary geometries, or the use of hybrid optimization methods to further improve robustness and computational efficiency.

In the future, we plan to extend our research to a more theoretical analysis of the problem, inspired by the [29,51,52]. We also plan to extend our approach by employing a two-sided spatial operator [53,54], which will allow us to account for the entire spatial domain. Finally, motivated by very recent developments in the design of numerical boundary conditions for fractional differential equations, we plan to perform a comparative study between the fractional Robin boundary conditions considered in this work and alternative approaches, such as the numerical anti-reflective boundary conditions introduced in [55]. Such a comparison, from both numerical and computational linear algebra perspectives, will allow us to better assess the impact of different boundary treatments on accuracy and stability.

## Figures and Tables

**Table 1:** Errors of approximate solution for direct problem.

Mesh (x,t)	$E_{\mathrm{mean}}$	$E_{\max}$	CPU Time [ms]
10×10	$1.411\times 10^{-1}$	1.073	52
100×100	$5.759\times 10^{-3}$	$1.079\times 10^{-1}$	467
100×200	$4.931\times 10^{-3}$	$1.078\times 10^{-1}$	1371
100×300	$4.704\times 10^{-3}$	$1.078\times 10^{-1}$	1868
100×500	$4.551\times 10^{-3}$	$1.078\times 10^{-1}$	3407
200×100	$2.943\times 10^{-3}$	$5.411\times 10^{-2}$	2181
300×100	$2.224\times 10^{-3}$	$3.611\times 10^{-2}$	5830
500×100	$1.758\times 10^{-3}$	$2.169\times 10^{-2}$	23,488
300×300	$1.176\times 10^{-3}$	$3.604\times 10^{-2}$	17,081
500×500	$5.592\times 10^{-4}$	$2.163\times 10^{-2}$	124,299

**Table 2:** Observed order of accuracy with respect to the spatial discretization ($N_{t}=100$).

Mesh (x,t)	$E_{\mathrm{mean}}$	Order
100×100	$5.759\times 10^{-3}$	–
200×100	$2.943\times 10^{-3}$	0.97
300×100	$2.224\times 10^{-3}$	0.69
500×100	$1.758\times 10^{-3}$	0.46

**Table 3:** Observed order of accuracy with respect to the temporal discretization ($N_{x}=100$).

Mesh (x,t)	$E_{\mathrm{mean}}$	Order
100×100	$5.759\times 10^{-3}$	–
100×200	$4.931\times 10^{-3}$	0.22
100×300	$4.704\times 10^{-3}$	0.12
100×500	$4.551\times 10^{-3}$	0.06

**Table 4:** Identification results in case of $x_{p}=1$; ${\bar{a}}_{i}$—reconstructed value of $a_{i}$ coefficient; $\Delta_{{\bar{a}}_{i}}$—absolute error of identification coefficient $a_{i}$; $\delta_{{\bar{a}}_{i}}$—relative error of identification coefficient $a_{i}$; *J*—the value of fitness function; σ—standard deviation of fitness function.

Noise	${\bar{a}}_{i}$	$\Delta_{{\bar{a}}_{i}}$	$\delta_{{\bar{a}}_{i}}\;\left[\%\right]$	*J*	$\sigma_{J}$
0%	$a_{0}=3.011$	0.00199	0.06		
	$a_{1}=-53.226$	0.0524	0.09		
	$a_{2}=340.061$	0.547	0.16	0.0256	0.1148
	$a_{3}=-21.854$	1.854	9.27		
	$a_{4}=151.103$	1.103	0.73		
1%	$a_{0}=3.004$	0.00501	0.16		
	$a_{1}=-53.043$	0.1306	0.24		
	$a_{2}=338.298$	1.216	0.35	21.9772	0.0714
	$a_{3}=-15.077$	4.923	24.61		
	$a_{4}=145.451$	4.549	3.03		
5%	$a_{0}=3.0194$	0.01039	0.34		
	$a_{1}=-53.417$	0.2434	0.45		
	$a_{2}=340.345$	0.831	0.24	377.3631	2.2351
	$a_{3}=-13.380$	6.62	33.1		
	$a_{4}=128.013$	21.987	14.65		
10%	$a_{0}=3.006$	0.00301	0.10		
	$a_{1}=-53.212$	0.0384	0.07		
	$a_{2}=341.043$	1.529	0.45	1681.8047	1.8192
	$a_{3}=-23.631$	3.631	18.15		
	$a_{4}=144.369$	5.631	3.75		

**Table 5:** Errors of estimation ψ function for the case of $x_{p}=1$: $E_{abs}$—absolute error of ψ estimation; $E_{rel}$—relative error of ψ estimation.

Noise	$E_{\mathrm{abs}}$	$E_{\mathrm{rel}}\;\left[\%\right]$
0%	24.294	0.041
1%	33.537	0.057
5%	32.523	0.055
10%	63.886	0.108

**Table 6:** Identification results in the case of $x_{p}=0.8$: ${\bar{a}}_{i}$—reconstructed value of $a_{i}$ coefficient; $\Delta_{{\bar{a}}_{i}}$—absolute error of identification coefficient $a_{i}$; $\delta_{{\bar{a}}_{i}}$—relative error of identification coefficient $a_{i}$; *J*—the value of fitness function; σ—standard deviation of fitness function.

Noise	${\bar{a}}_{i}$	$\Delta_{{\bar{a}}_{i}}$	$\delta_{{\bar{a}}_{i}}\;\left[\%\right]$	*J*	$\sigma_{J}$
0%	$a_{0}=3.010$	0.00099	0.03		
	$a_{1}=-53.212$	0.0384	0.07		
	$a_{2}=339.861$	0.347	0.10	0.0226	0.2773
	$a_{3}=-20.860$	0.86	4.30		
	$a_{4}=149.550$	0.45	0.30		
1%	$a_{0}=3.004$	0.00501	0.16		
	$a_{1}=-53.018$	0.1556	0.29		
	$a_{2}=337.701$	1.813	0.53	7.1458	0.1428
	$a_{3}=-11.417$	8.583	42.91		
	$a_{4}=138.201$	11.799	7.86		
5%	$a_{0}=2.996$	0.01301	0.43		
	$a_{1}=-52.901$	0.2726	0.51		
	$a_{2}=338.443$	1.071	0.31	192.2048	0.2685
	$a_{3}=-24.653$	4.653	23.26		
	$a_{4}=158.945$	8.945	5.96		
10%	$a_{0}=2.991$	0.01801	0.59		
	$a_{1}=-52.526$	0.6476	1.21		
	$a_{2}=333.666$	5.848	1.72	703.0846	1.4075
	$a_{3}=-11.277$	8.723	43.61		
	$a_{4}=153.642$	3.642	2.42		

**Table 7:** Identification results in the case of $x_{p}=0.5$: ${\bar{a}}_{i}$—reconstructed value of $a_{i}$ coefficient; $\Delta_{{\bar{a}}_{i}}$—absolute error of identification coefficient $a_{i}$; $\delta_{{\bar{a}}_{i}}$—relative error of identification coefficient $a_{i}$; *J*—the value of fitness function; σ—standard deviation of fitness function.

Noise	${\bar{a}}_{i}$	$\Delta_{{\bar{a}}_{i}}$	$\delta_{{\bar{a}}_{i}}\;\left[\%\right]$	*J*	$\sigma_{J}$
0%	$a_{0}=3.006$	0.00301	0.10		
	$a_{1}=-53.064$	0.1096	0.20		
	$a_{2}=338.115$	1.399	0.41	0.0102	0.0938
	$a_{3}=-13.751$	6.249	31.245		
	$a_{4}=141.389$	8.611	5.74		
1%	$a_{0}=3.012$	0.00299	0.09		
	$a_{1}=-53.299$	0.1254	0.23		
	$a_{2}=341.025$	1.511	0.44	1.9317	0.1589
	$a_{3}=-25.415$	5.415	27.07		
	$a_{4}=153.954$	3.954	2.63		
5%	$a_{0}=3.013$	0.00399	0.13		
	$a_{1}=-52.901$	0.2254	0.42		
	$a_{2}=343.108$	3.594	1.05	61.6646	1.3642
	$a_{3}=-37.355$	17.335	86.76		
	$a_{4}=167.019$	17.019	11.34		
10%	$a_{0}=3.032$	0.02299	0.76		
	$a_{1}=-53.905$	0.7314	1.37		
	$a_{2}=345.334$	5.820	1.71	201.6490	0.0310
	$a_{3}=-26.506$	6.506	32.53		
	$a_{4}=125.233$	24.767	16.51		

**Table 8:** Errors in estimation ψ function for the case of $x_{p}=0.8$ and $x_{p}=0.5$ measurement point: $E_{abs}$—absolute error of ψ estimation; $E_{rel}$—relative error of ψ estimation.

Noise	$x_{p}=0.5$		$x_{p}=0.8$	
	$E_{\mathrm{abs}}$	$E_{\mathrm{rel}}\;\left[\%\right]$	$E_{\mathrm{abs}}$	$E_{\mathrm{rel}}\;\left[\%\right]$
0%	14.919	0.025	12.596	0.021
1%	10.516	0.018	29.627	0.051
5%	1589.550	2.735	88.966	0.153
10%	66.572	0.114	80.249	0.138

## Data Availability

The original contributions presented in this study are included in the article. Further inquiries can be directed to the corresponding author.
